# Supplementary material for: A set of multi-entry identification keys to African frugivorous flies (Diptera, Tephritidae)
Source: Zookeys. 2014 Jul 24;(428):97–108. doi: 10.3897/zookeys.428.7366 (PMC4143993; doi:10.3897/zookeys.428.7366)
Supplement: Supplementary material 6 — Key to Ceratitis [file zookeys-428-097-s006.zip › SF6_ZooKeys_key to Ceratitis/key/SF6_ZooKeys_key to Ceratitis/Media/Html/Ceratitis andranotobaka .htm]

Ceratitis (Ceratalaspis) andranotobaka Hancock


***Ceratitis*** **(*Ceratalaspis*)
*andranotobaka* Hancock**

*Ceratitis andranotobaka* Hancock, 1984: 285.

Body
length: 4.40 mm; wing length: 4.50 mm.

Male

Head.
Antenna yellow-orange. Third antennal segment two times as long as second
segment. Arista with short hairs over entire length. Frons yellow to orange;
with short scattered hairs which are distinctly darker than the frons; in
lateral view not distinctly projecting forwards at antennal implant. Lower eye
margin with darker colouration. Chaetotaxy normal for subgenus.

Thorax.
Postpronotum pale yellow, unspotted, only slightly paler than centre of
mesonotum,. Ground colour of mesonotum pale with yellow-orange tinge.
Chaetotaxy normal for subgenus. Scapular setae black. Two anepisternal
bristles. Scutellum mainly yellow; with three black separate markings apically
except at ventral margin where narrowly joining; basally two dark distinct
spots. Subscutellum pale with three dark separate spots except along dorsal
edge where touching.

Legs.
Yellow; setation typical for subgenus, mainly pale, especially on femora.
Posterior and posterodorsal rows on fore femur pale.

Wing.
Bands with markings extensively yellow. Banding, marginal band continuous;
cubital band free; medial band joined with marginal band; crossvein r-m at middle
of discal cell. Crossvein dm-cu posteriorly more inwards than anteriorly.

Abdomen.
Orange-brown. Setation and banding typical for subgenus.

Female

As
male except for the following characters: third antennal segment more orange
coloured. Three frontal bristles. Inner scapular setae pale. Medial band
interrupted in the middle. Oviscape as long as abdominal terga 3-6 combined.

(Description
after De Meyer, 1998)
